# T cells but not NK cells are associated with a favourable outcome for resected colorectal liver metastases

**DOI:** 10.1186/1471-2407-14-180

**Published:** 2014-03-13

**Authors:** Siân A Pugh, Rebecca J Harrison, John N Primrose, Salim I Khakoo

**Affiliations:** 1 University Surgery, Southampton General Hospital, Southampton, UK; 2 Department of Hepatology, Southampton General Hospital, Southampton, UK

**Keywords:** Natural Killer cells, Colorectal liver metastases, Innate immunity, Colorectal cancer, Adaptive immunity, T cells, CD56+ T cells

## Abstract

**Background:**

The adaptive immune response to colorectal cancer is important for survival. Less is understood about the role of innate lymphocytes, such as Natural Killer (NK) cells, which are abundant in human liver.

**Methods:**

Samples of fresh liver (n = 21) and tumour (n = 11) tissue were obtained from patients undergoing surgical resection of colorectal liver metastases. Flow cytometry was used to analyse the presence and phenotype of NK cells, as compared to T cells, in the tumour and liver tissue. Results were correlated with survival.

**Results:**

NK cells were poorly recruited to the tumours (distant liver tissue 38.3%, peritumoural liver 34.2%, tumour 12.9%, p = 0.0068). Intrahepatic and intratumoural NK cells were KIR (killer immunoglobulin-like receptor)^lo^NKG2A^hi^ whereas circulating NK cells were KIR^hi^NKG2A^lo^. By contrast T cells represented 65.7% of the tumour infiltrating lymphocytes. Overall survival was 43% at 5 years, with the 5-year survival for individuals with a T cell rich infiltrate being 60% (95% CI 17-93%) and for those with a low T cell infiltrate being 0% (95% CI 0-48%). Conversely individuals with higher levels of NK cells in the tumour had an inferior outcome, although there were insufficient numbers to reach significance (median survivals: NK^Hi^ 1.63 years vs NK^Lo^ 3.92 years).

**Conclusions:**

T cells, but not NK cells, are preferentially recruited to colorectal liver metastases. NK cells within colorectal metastases have an intrahepatic and potentially tolerogenic, rather than a peripheral, phenotype. Similar to primary tumours, the magnitude of the T cell infiltrate in colorectal metastases is positively associated with survival.

## Background

The liver is the most common site of metastasis of colorectal cancer. When surgical resection of liver metastases is possible, the median five-year survival now approaches 40% [1]. For those who do not survive this is almost always due to disease recurrence. A number of factors are known to influence the prognosis of patients with colorectal liver metastases such as stage of primary disease, synchronous presentation, number of metastases, and carcinoembryonic antigen level [2,3]. There is also increasing evidence that the host response to colorectal cancer is relevant to disease progression and survival [4-8]. Whilst the majority of such evidence concerns stage I-III disease, there is emerging data, albeit limited, for the role of immunosurveillance in colorectal liver metastases [9-11]. One study reported more CD8 T cells and less CD4 T cells in the metastases of patients surviving ≥10 years post liver resection compared to ≤2 year survivors [9]. In addition there is evidence that a high number of regulatory T cells relative to CD4 or CD8 T cells is predictive of a poor outcome which is in accordance with data from primary colorectal cancer [10,11].

To date it is primarily cells of the adaptive immune system that are understood to be key in determining outcome from colorectal cancer [4,6,8,10-12]. The liver however has a unique immunological environment in which the lymphoid population is weighted with innate immune cells [12,13]. Within the liver Natural Killer (NK) cells are the predominant innate lymphocyte population accounting for up to 50% of human hepatic lymphocytes compared to less than 20% of the lymphocytes in peripheral blood [14-16]. These lymphocytes are potent anti-tumour effector cells both through an ability to directly kill target cells without the need for prior sensitisation, and also secrete cytokines that influence the adaptive immune response [17]. In addition the liver has a high prevalence of CD56^+^ T cells [13,15]. These CD56^+^CD3^+^ lymphocytes are also capable of mediating target cell lysis in the absence of prior immunisation with antigen.

The relative abundance of innate lymphocytes in human liver should represent a significant defence to hepatic malignancy. However, as a site of early encounter of antigens the liver must be tolerogenic to dietary antigens and commensal organisms at the same time as being able to mount an effective immune response to pathogens or tumour cells [18,19]. As such it is thought that hepatic tolerance may be favoured over the induction of immunity. Indeed allogeneic liver transplants across fully incompatible HLA (human leukocyte antigen) barriers sometimes survive without the need for immunosuppression [20].

This study sought to determine the relative contribution of innate versus adaptive immunity to the hepatic defence against colorectal metastasis. The relative recruitment of Natural Killer cells, CD56^+^ T cells and CD56^−^ T cells to colorectal liver metastases was analysed and correlated with long-term survival. This was determined using flow cytometry to allow accurate analysis of the lymphocyte subsets in fresh tissue collected from patients undergoing resection of metastatic disease in the liver.

## Methods

### Study population

A total of 21 patients undergoing surgical resection of their liver for colorectal metastasis at Southampton General Hospital, Southampton, UK between July 2006 and October 2007 were included. Patients were between 40 and 80 years of age and 13 were male. Ethical permission was obtained from the Southampton and South West Hampshire Research Ethics Committee. Written informed consent was obtained from all participants. At a median follow up of 6.5 years, 12 patients were still alive, eight had died of disease and one died of transitional cell carcinoma of the bladder. The median survival for the whole cohort was 3.92 years with a five year overall survival of 43% (95% CI 23%-66%). An additional 17 healthy controls, 10 male mean age 37 years, gave consent for the use of peripheral blood and were included.

### Isolation of lymphocytes from liver/tumour tissue

Samples of fresh liver only (n = 21) and paired liver and tumour tissue (n = 11) were obtained following surgical resection, placed in liver solution, [21] and processed immediately. These were macroscopically divided into tumour tissue, peritumoural liver tissue (defined as the liver tissue immediately adjacent to the tumour) and liver tissue distant from the tumour site (<5 cm from the tumour). The tissue was dissected into approximately 2 mm^3^ fragments and the total mass recorded for later determination of the number of mononuclear cells/mg of tissue. Following this, collagenase type IV (Sigma, Poole, UK) and DNase I (Sigma, Poole, UK) at final concentrations of 0.05% and 0.002% respectively were added to the dissected tissue. Samples were incubated at 37°C for 30 minutes on an orbital shaker. Undissociated tissue was removed by filtering the samples through a 70 μm cell strainer (Becton Dickinson, Oxford, UK). Ficoll density gradient centrifugation was used to isolate the mononuclear cells followed by trypan blue exclusion to count the live cells.

In order to ascertain that this process of enzymatic digestion did not result in a loss of phenotypic markers this method was directly compared to a purely mechanical method of dissociation. Not only did the enzymatic method yield consistently higher numbers of mononuclear cells, flow cytometric analysis confirmed that there was no loss of phenotypic marker expression (data not shown).

### Isolation of mononuclear cells from whole blood

Samples of matched blood were obtained from seven patients prior to surgery. Blood was diluted 1:1 with RPMI and layered on an equal volume of Ficoll for density gradient centrifugation. Trypan blue exclusion was used as described above.

### Flow cytometry

Immunophenotypic analysis was performed using three colour flow cytometry on a FACS Calibur (Becton Dickinson) and six colour flow cytometry on a FACS Canto (Becton Dickinson). Antibodies used were CD45 FITC, 2DL2/3 FITC, CD56 PE, NKG2D PE, CD3 PerCP, Streptavidin PerCP, CD56 PE-Cy7, 2DL1 APC, CD56 APC, CD3 APC-Cy7, 3DL1 biotin (all Becton Dickinson). Ig isotype controls were used where appropriate. Lymphocyte gating was performed using the forward scatter versus side scatter plot and 10 000 events were acquired. NK cells were identified as CD3^−^CD56^+^, T cells as CD3^+^CD56^−^, and CD56^+^ T cells as CD3^+^CD56^+^. Data was analysed using Cell Quest/FACS Diva software.

### Statistical methods

Statistical analyses were performed using GraphPad Prism version 5 (GraphPad, La Jolla, CA, USA). Comparisons were made using non-parametric tests (Mann–Whitney *U*-test and Kruskal Wallis test) to assess differences in the expression of phenotypic markers by lymphocytes isolated from liver, tumour and peripheral blood. Kaplan-Meier plots were analysed using the log-rank test.

## Results

**Figure 1:**
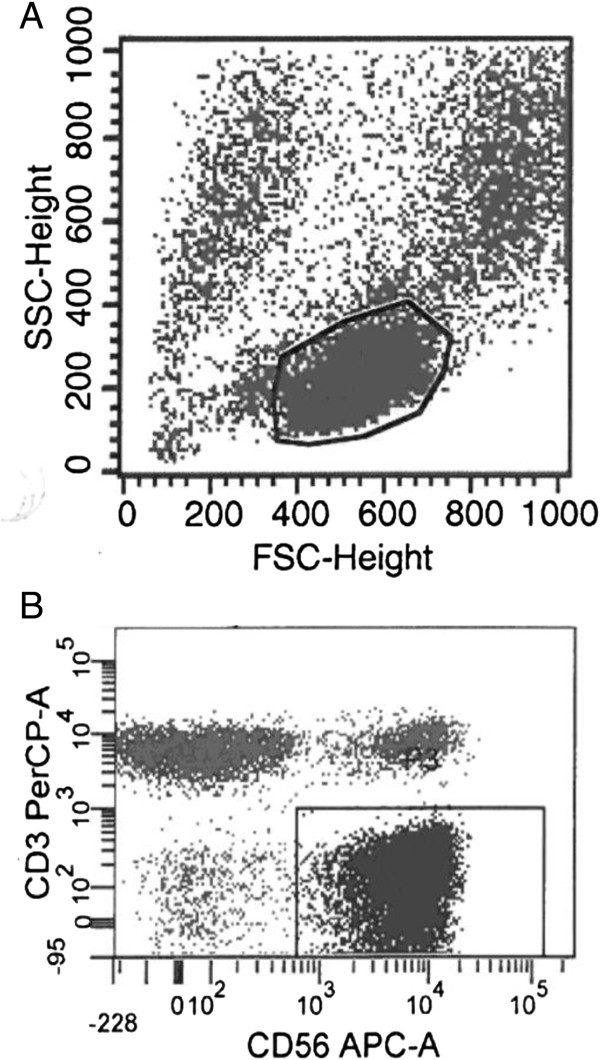
**Flow cytometry was used to determine the subsets of lymphocytes present in liver, tumour and peripheral blood.** Shown are two dot plots demonstrating the gating using forward scatter and side scatter **(A)**, and CD56 versus CD3 **(B)** demonstrating the populations of NK cells, T cells, and CD56+ T cells.

**Figure 2:**
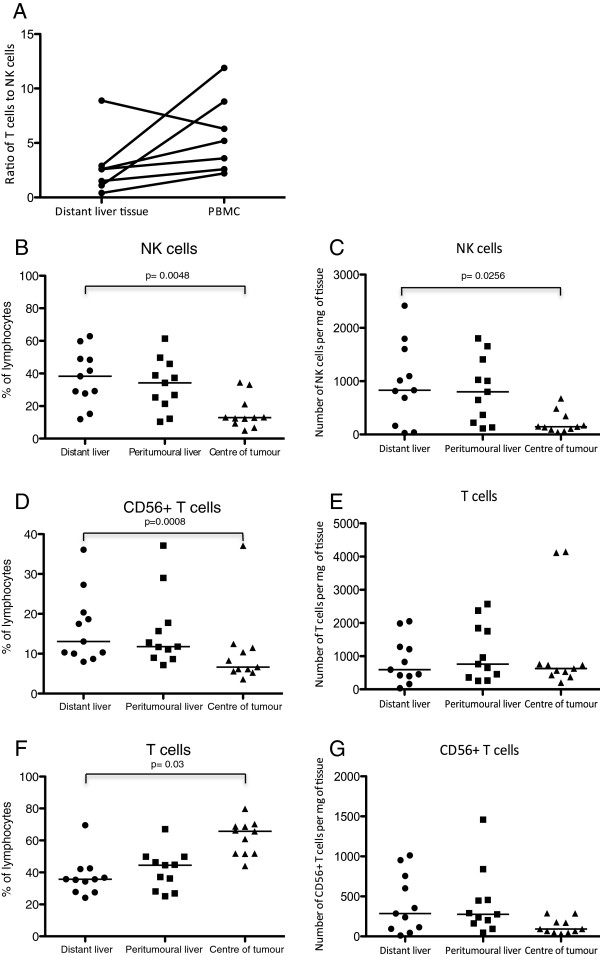
**Liver tissue, tumour tissue and peripheral blood samples were analysed for the presence of NK cells (CD56**^**+**^**CD3**^**−**^**), T cells (CD3**^**+**^**CD56**^**−**^**) and CD56**^**+ **^**T cells (CD56**^**+**^**CD3**^**+**^**) by flow cytometry. A** The ratio of T cells to NK cells in distant liver tissue and peripheral blood (PBMC) is presented for seven patients in whom matched blood samples were obtained. The percentages **(B**, **D**, **F)** and actual numbers **(C**, **E**, **G)** of NK cells (CD56^+^CD3^−^), T cells (CD3^+^CD56^−^), and CD56^+^ T cells (CD56^+^CD3^+^) in the tumour, peritumoural liver and distant liver tissue were determined in 11 patients.

We evaluated the frequencies of NK cells, T cells and CD56^+^ T cells in the macroscopically unaffected (“distant”) liver, the peritumoural liver and within the tumour tissue itself using flow cytometry (Figure 1). Consistent with previous reports and of the model that the liver is an innate organ there were fewer CD56^−^ T cells (42.5% vs 62.2% p = 0.018), but more CD56^+^ T cells (21% vs 4.9%, p = 0.053) and CD56^+^ NK cells (19.7% vs 13.6% p = ns) in the macroscopically unaffected liver than peripheral blood. As such there was a higher ratio of T cells to NK cells in peripheral blood as compared to liver tissue in all but one of seven patients (Figure 2A).

**Figure 3:**
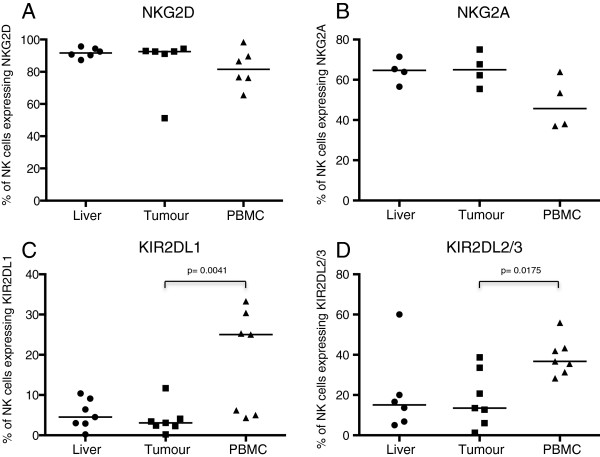
Expression of the NK cell markers NKG2D (A), NKG2A (B), KIR2DL1 (C) and KIR2DL2/3 (D) as determined by flow cytometry of NK cells isolated from liver tissue, tumour and PBMC.

Overall we found no difference in the frequencies of lymphocytes between the distant and peritumoural liver tissue (NK cells: distant liver 38%, peritumoural liver 34%, T cells: distant liver 36%, peritumoural liver 45%, CD56^+^ T cells: distant liver 13%, peritumoural liver 12%) (Figures 2B, D, F). However we noted that the lymphocyte population within the tumour was substantially different from the background liver, with an increased frequency of T cells (61.8% vs 37.4% p = 0.0008) and a depressed frequency of NK cells (12.9% vs 38.3% p = 0.0048) and CD56^+^ T cells (6.7% vs 13.1% p =0.03). Furthermore the absolute number of CD56^−^ T cells was similar at all three sites sampled (macroscopically normal liver tissue, 595 cells/mg, peritumoural liver 760 cells/mg and tumour 626 cells/mg p = ns), whilst the intratumoral NK cell number was significantly lower than the macroscopically normal tissue (145 cells/mg vs 832 cells/mg p = 0.026) and there was a trend towards a lower number of CD56^+^ T cells in the tumour (92 cells/mg) compared to the macroscopically normal liver (285 cells/mg) (Figures 2C, E, G). Thus there appears to be a preferential recruitment of T cells, rather than NK cells or CD56+ T cells to the tumour. To investigate further the relative paucity of NK cells within the tumour we phenotyped them in more detail. This showed that the intratumoural NK cells have an expression profile more similar to that found in the intrahepatic, rather than peripheral blood compartment. In particular NK cells in the liver and the tumour were relatively KIR (killer immunoglobulin-like receptor) ^lo^ (median% of NK cells expressing KIR2DL1 and KIR2DL2/3 in: liver 4.5% and 15.1%, tumour 3.1% and 13.5%, peripheral blood 25% and 36.7%) and NKG2A^hi^ (median% of NK cells expressing NKG2A: liver 65%, tumour 65%, peripheral blood 46%) when compared with NK cells isolated from peripheral blood (Figure 3). Thus either there is a modest influx of NK cells from the liver, or the tumour microenvironment is more conducive to recruitment of NKG2A^hi^ NK cells.

**Figure 4:**
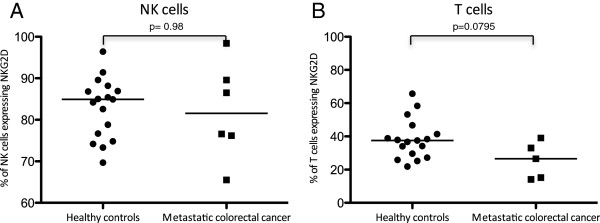
NKG2D expression by NK cells (A) and T cells (B) from PBMC of seven patients with colorectal liver metastases and 17 healthy controls as determined by flow cytometry.

NKG2D is an activating NK cell receptor, also expressed by cytotoxic T cells, that is downregulated in the presence of colorectal cancer due to the shedding of MIC-A/B by the tumour [22,23]. This may be one mechanism of immune evasion by colorectal cancer. However NKG2D expression was maintained on NK cells isolated from peripheral blood of patients with colorectal liver metastases (median% of NK cells expressing NKG2D from peripheral blood of patients with metastases 81.6% vs healthy volunteers 84.9%, p = 0.98), and also on the tumour infiltrating NK cells (median 92.6%) indicating that NKG2D downregulation was not a cause for the weak NK cell response (Figure 4).

**Figure 5:**
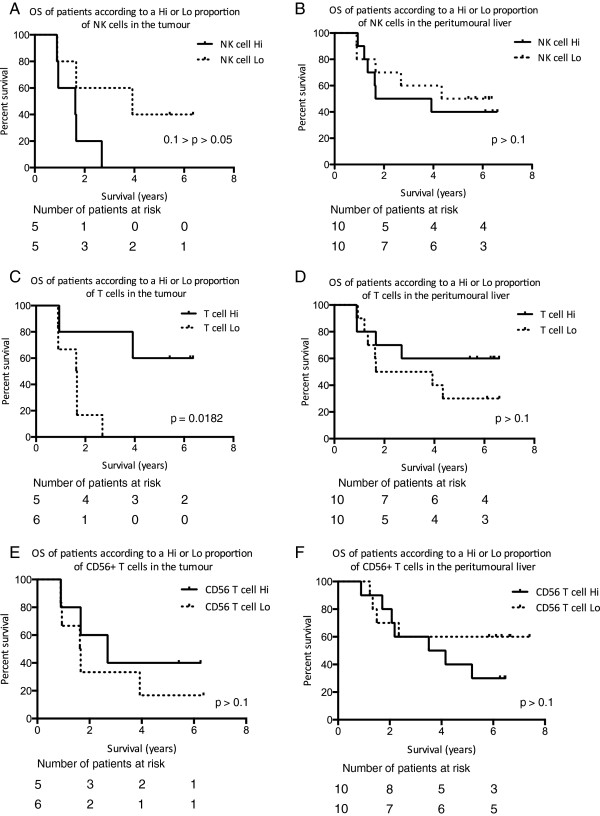
**Kaplan-Meier plots of overall survival of patients according to Hi (greater than or equal to the median) or Lo (lower than the median) proportions of NK cells (CD56**^**+**^**CD3**^**−**^**) (A, B), T cells (CD56**^**−**^**CD3**^**+**^**) (C, D), or CD56**^**+ **^**T (CD56**^**+**^**CD3**^**+**^**) cells (E, F) in the tumour of 11 patients and peritumoral liver tissue of 20 patients.**

In order to understand whether the composition of the intratumoural lymphocyte infiltrate had an impact on outcome we divided the patients into two groups “Hi” and “Lo” depending on whether the lymphocyte composition was greater or less than the median value of the population. Patients were followed up for a minimum of 5.8 years and the outcome data correlated to the lymphocyte frequencies (Figure 5). Patients with a T cell rich intratumoural infiltrate had improved survival as compared to those with low levels of T cell infiltration (p = 0.0182 Figure 5C). Conversely those with a low NK cell infiltrate had improved survival. There was no influence of peritumoural lymphocytes on outcome. Previous studies have utilised immunohistochemical based analyses and as such calculated numbers of lymphocytes rather than frequencies, however, we found no correlation between absolute numbers of T cells or NK cells and survival (data not shown). Overall, our data suggest that a strong T cell, but not NK cell or CD56^+^ T cell response to the tumour is associated with a favourable prognosis.

## Discussion

The normal liver must remain tolerogenic to a multitude of harmless antigens from the gut whilst retaining vigilance to toxins and tumour cells [18,19]. This results in a unique immunological environment rich in cells of the innate immune system that includes a high proportion of NK cells and CD56^+^ T cells [13,14]. NK cells are lymphocytes originally defined based on their prompt spontaneous killing of malignant cells [24]. This relative abundance of NK cells and CD56^+^ T cells, both with potent anti-tumour functions, should represent a substantial defence against metastasis to the liver. Indeed our initial hypothesis was that there would be a gradient across the liver, between distant and peritumoural liver tissue, indicative of migration of hepatic NK cells towards the tumour. Whilst our findings confirm that livers containing colorectal metastases are relatively rich in these innate lymphocyte subsets, these data suggest that they contribute little to the immunological defence against these tumours since no evidence was found of migration and there was a paucity of intratumoural NK and CD56^+^ T cells. In our initial studies we also hypothesised that invariant NKT cells expressing the receptor Vα24Jα18 may be involved in the response to hepatic malignancy. However they were present in frequencies lower than 1% (data not shown), consistent with the observations of others, [25] and therefore we did not pursue this further.

Interestingly the few NK cells that were isolated from the tumour tissue had a similar phenotype to those in the macroscopically normal liver, rather than the peripheral blood, being NKG2A^hi^ and KIR^lo^. This suggests that the tumour has “escaped” the intrahepatic NK cell response consistent with the model that there is immune editing of the tumour within the liver.

Our observations that a higher infiltrate of T cells (CD3^+^CD56^−^) to the tumour correlates positively with an improved survival is in concordance with published data in both primary colorectal cancer and colorectal liver metastases using immunohistochemistry [4,8-10]. In this study we utilised flow cytometry, instead of the commonly used immunohistochemical analysis of FFPE tissue, in order to examine the lymphocyte subsets present in fresh liver and tumour tissue. This allowed the analysis of much larger quantities of tissue avoiding the potential sampling bias due to heterogeneity of the lymphocytic infiltrate. Furthermore the use of fresh tissue taken at the time of resection enabled pieces of specimen to be selected, such as peritumoural and distant liver tissue, rather than being limited by stored tissue. In fact we observed that the intratumoural as opposed to the peritumoural liver T lymphocyte response is important in determining outcome. Data from primary colorectal cancer has shown both the lymphocyte infiltrate at the invasive margin as well as the centre of the tumour to correlate with survival [4]. However in our study peritumoural liver was examined since liver metastases tend to have what is described as a pushing margin rather than an invasive margin. The finding that higher NK cell levels are associated with a worse outcome is consistent with recent reports of NK cell “fratricide” of antigen specific T cells and thus can attenuate adaptive immunity [26,27].

It has been shown by a number of authors that a higher number of T cells infiltrating the tumour is a predictor of superior survival. This benefit does however appear to be dependent upon the relative proportions of T cell subsets present, with a higher number of regulatory T cells proportional to CD4 or CD8 T cells predictive of a worse outcome [10]. In this study we did not analyse the T cell subsets, other than to look at CD56^+^ T cells, the proportion of which did not correlate with survival. These are an innate sub-population of T cells which are relatively enriched in the liver. The absence of an association of these lymphocytes with outcome is consistent with our observation that the intrahepatic lymphocyte microenvironment does not appear to play a significant role in the immune response to colorectal liver metastasis.

We have shown that NK cells poorly infiltrate the tumour as compared with other lymphocytes such as T cells. The phenotypic similarities between the hepatic and intratumoural NK cells may suggest the NK cells that do infiltrate the tumour are derived from the hepatic population. What we now understand, both from our own and others data, is that these hepatic NK cells are functionally distinct from circulating NK cells [28]. Hepatic NK cells have decreased cytotoxic function both because of a higher percentage of CD56^bright^ (CD16^−^) NK cells and overall decreased cytolytic activity of the CD56^dim^ (CD16^+^) NK cells. One hypothesis is that this is due to fewer licensed NK cells within the liver thereby resulting in a functionally hyporesponsive population.

It is perhaps more challenging to acknowledge that those patients with a higher infiltrate of NK cells to the tumour appear to have an inferior outcome following resection. However, given that the liver is continuously confronted with a large antigenic load, much of which is harmless, immune responses must be carefully regulated and it appears that there is an overall tendency towards tolerance rather than immunity. Therefore rather than hepatic NK cells providing a strong anti-tumour defence, it may be that this “tolerant” population contributes to the susceptibility of the liver to metastatic disease.

## Conclusion

In conclusion we have shown that despite a relative abundance of NK cells in the liver, there is a preferential recruitment of T cells rather than NK cells to colorectal liver metastases. This poor infiltration, together with the trend suggesting a negative correlation with survival, likely reflects a tendency towards tolerance of hepatic NK cells rather than immunity. By contrast there is a strong association between T cell infiltration to the tumour and survival post liver resection. If this can be validated in a larger cohort of patients it may be utilised to predict likelihood of recurrence following resection of colorectal liver metastases with curative intent.

## Competing interests

The authors declare that they have no competing interests.

## Authors’ contributions

SP participated in the design of the study, carried out the laboratory work, completed the analysis and drafted the manuscript. RH carried out the flow cytometric analysis on blood from healthy controls. JP participated in the design of the study, facilitated the provision of fresh tissue, and helped draft the manuscript. SK conceived of the study, participated in the design, assisted with the analysis and helped to draft the manuscript. All authors read and approved the final manuscript.

## Pre-publication history

The pre-publication history for this paper can be accessed here:

http://www.biomedcentral.com/1471-2407/14/180/prepub
